# Supplementary material for: Towards unified AI-driven fracture mechanics: the extended deep energy method (XDEM)
Source: Nat Commun. 2026 Aug 15;17:8492. doi: 10.1038/s41467-026-76748-1 (PMC13484471; doi:10.1038/s41467-026-76748-1)
Supplement: Supplementary file 3 [file 41467_2026_76748_MOESM3_ESM.zip › pic/Results/Stress_intensity_factor/Mixed/beta_15/crack_gamma_beta15.pdf]

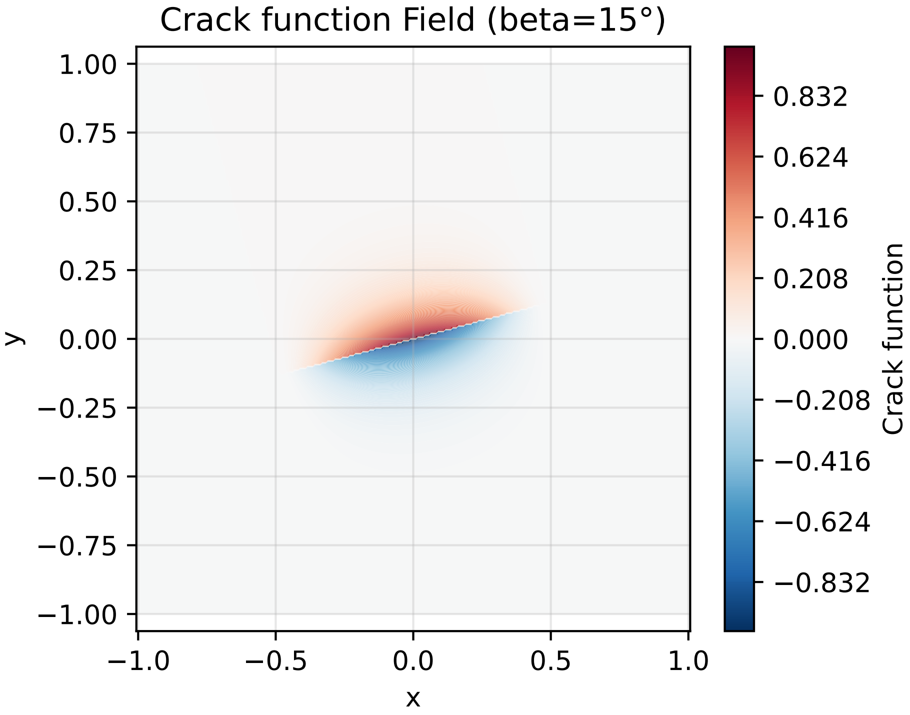

Crack function Field (beta=15°)
1.00
0.75
0.50
0.25
0.00
−0.25
−0.50
−0.75
−1.00
y
−1.0
−0.5
0.0
0.5
1.0
x
0.832
0.624
0.416
0.208
0.000
−0.208
−0.416
−0.624
−0.832
Crack function
